# Lineup Types and Key Variables for Success in Men's Paralympic Wheelchair Basketball

**DOI:** 10.5114/jhk/210187

**Published:** 2025-11-20

**Authors:** William Becerra-Muñoz, Javier Pérez-Tejero

**Affiliations:** 1 “Fundación Sanitas” Chair for Inclusive Sport Studies (CEDI), AFIPE Research Group, Department of Health and Human Performance, Faculty of Physical Activity and Sport Sciences, Universidad Politécnica de Madrid, Spain.

**Keywords:** performance analysis, game-related statistics, paralympic sport, team performance

## Abstract

Understanding team performance dynamics in wheelchair basketball (WB) is crucial for optimising lineup compositions for different game situations. This study aimed to investigate the impact of lineup composition and game time on performance in men's WB at the Tokyo 2020 Paralympic Games. Through the analysis of 588 lineups from 42 matches, key game-related statistics (GRS) that differentiated winning from losing lineups were identified. Seventy different lineup types (LTs) were clustered and categorised based on players’ functional classification (FC) and their performance across time windows was examined. Discriminant analysis revealed that field goal efficiency, assists, and minimising opponent assists were crucial for success, regardless of game balance, emphasising the importance of both offensive and defensive efficacy. Clustering categorised LTs into three distinct groups, with those featuring predominantly intermediate-point players (2.0–3.5) exhibiting superior performance, particularly during extended playing time (over 10 minutes), indicating a strategic advantage for these compositions in sustaining high-level play. Top-ranked teams typically showed greater roster depth and a wider variation of LT utilisation. This research highlights the significance of strategic lineup composition, emphasising the sustained effectiveness of lineups with intermediate FC players. The findings provide valuable insights for coaches, aiding in data-driven decisions for lineup selection, game strategy optimisation, and functional player development within the specific demands of WB.

## Introduction

Wheelchair basketball (WB) is a prominent sport played globally by individuals with physical disabilities, including spinal cord injuries and lower limb amputations mainly. It is estimated that around 100,000 players practise this sport in more than 100 countries across the world (IWBF, 2021a). The two most notable features of this sport are the use of sport-specific wheelchairs and the use of a functional classification (FC) system for players. FC of players in WB ranges from 1.0 to 4.5 points, based on their degree of functional movement capacity using the wheelchair and evidenced during the game (IWBF, 2021b). Notably, this classification system comprises eight specific classes, each separated by 0.5 points, allowing for detailed differentiation of player abilities. The system considers the player's ability to execute fundamental technical actions, such as pushing the wheelchair, braking, turning, dribbling, moving forward, and shooting at the basket (Vanlandewijck et al., 2001).

Players classified as 1.0 and 1.5 points exhibit the most significant functional limitations, with minimal trunk control and restricted upper extremity movements, typically functioning as agile guards in low-height wheelchairs (IWBF, 2021b). Class 2.0-point players demonstrate improved trunk control, enabling lateral and forward inclinations, enhancing wheelchair handling and speed, though limited in wide turns (IWBF, 2021b; Vanlandewijck et al., 2011; Wang et al., 2005). Class 3.0-point players show a marked increase in trunk control, facilitating multidirectional movements and dynamic balance, making them versatile forwards combining offensive and defensive duties, with player height influencing their role (Iturricastillo et al., 2022; Wang et al., 2005). Players in classes 4.0 and 4.5, demonstrate advanced or near-complete trunk control, respectively (IWBF, 2021b), excel in internal positions like centres or power forwards, with 4.5-point players demonstrating superior rebounding and close-basket finishing (Pérez-Tejero and Pinilla-Arbex, 2015). Findings have consistently demonstrated an incremental relationship between FC and game performance, with higher FC players generally exhibiting better game-related statistics (GRS), higher shooting efficiency for players with FC above 3.0, and greater efficiency in men's compared to women's competitions (Hernández-Beltrán et al., 2024; Molik et al., 2009; Pérez-Tejero and Pinilla-Arbex, 2015; Vanlandewijck et al., 2003, 2004). Specifically, 4.0- and 4.5-point players outperformed lower FC classes, with reduced performance differences observed between closely related classes.

This functional distribution shapes individual player capabilities and defines specific roles that optimise team performance, fostering tactical and strategic diversity (Francis et al., 2019; Vanlandewijck et al., 2001). During international competitions, lineup composition must adhere to the FC criterion, limiting the sum of the five on-court players' FCs to a maximum of 14 points (IWBF, 2021b). The FC criterion influences not only on-court lineup composition but also the selection of the full roster for a given competition.

Over the past two decades, researchers have utilised GRS to analyse players’ performance during international WB competitions, focusing on the relationship between FC and on-court performance. Studies aimed to determine how FC and the player’s position impact GRS (Vanlandewijck et al., 2003, 2004), to examine the incremental relationship between FC and individual performance (Molik et al., 2009; Pérez-Tejero and Pinilla-Arbex, 2015), to identify key GRS factors influencing game outcomes (Gómez et al., 2014, 2015), and to assess shooting efficiency based on FC and gender (Hernández-Beltrán et al., 2024). These investigations collectively pursued to understand the determinants of successful individual and team performance within the unique context of WB. They also highlighted the influence of team quality, playing time and FC on GRS (Gómez et al., 2015; Molik et al., 2009; Pérez-Tejero and Pinilla-Arbex, 2015). Additionally, higher team quality value correlated with better individual player performance in GRS. Although some studies have accounted for playing time as a variable (Gómez et al., 2015; Pérez-Tejero et al., 2020), it has primarily been analysed concerning physiological demands (Iturricastillo et al., 2018; Marszałek et al., 2019). In contrast to WB, in running basketball, performance analysis based on time windows has been considered, particularly in studies examining peak demands during competition over 1-, 5-, and 10-min periods (Alonso et al., 2020; Fox et al., 2021; Pérez-Chao et al., 2023). However, the influence of time considering lineup analysis in WB performance competition, remains unknown.

In contrast to individual player analysis, a team-centric perspective on WB performance analysis from GRS has also emerged in recent years. This is particularly relevant as athlete performance analysis is crucial for training and prediction in all sports (McLaren et al., 2018), and especially in team sports (Huyghe et al., 2022; Ibáñez et al., 2008), where players’ interactions and opponent opposition add complexity to the analysis (Balague et al., 2013; Pol et al., 2020). Research within this domain suggests that selecting optimal lineups that can score points and prevent the opponent from scoring through defensive pressure and maintaining a margin of points in favour of the result was found crucial in winning a game (Becerra-Muñoz et al., 2023; Francis et al., 2019). In addition, several studies have recently begun to focus on providing coaches with more information to optimise lineup composition. These approaches included clustering players based on game performance (high-performing or low-performing) to inform lineup composition (Cavedon et al., 2024), designing data-driven algorithms to generate lineups based on individual performance and FC (Calvo et al., 2024), and analysing tactical performance (Arroyo et al., 2023; Yasuda et al., 2024), lineup types (LT), their FC composition, and their performance during a given competition (Becerra-Muñoz et al., 2023). Whilst team-centric analysis provides initial insights into lineup composition, including FC prevalence and the most used LTs in winning teams, it remains limited in scope. Thus, further research is essential to understand the impact of specific LTs on team performance fully and to identify lineup trends across various game situations and time windows.

Therefore, this study had a triple objective: first, to determine the GRS that would best discriminate between winning and losing lineups at the Tokyo 2020 Paralympic Games male competition, in order to identify the key variables of winning a match at the elite level; second, to describe the composition of the classified LTs and to identify performance differences by game time; finally, to describe performance of the most-used lineups by the participating teams.

## Methods

### Sample

Official match statistics from the men's WB competition at the Tokyo 2020 Paralympic Games were obtained from the official website, specifically from the Line-Up Analysis report, where the team's performance (for each different five-player composition used on the court) at a given time during each match is provided. The sample comprised 588 lineups used across 42 matches played by the 12 national teams during the different phases of the competition: group stage (round-robin system by group) and playoff stage (quarterfinals, semifinals, and finals). The collected GRS included all game variables: difference between points made and received (plus/minus), field goals made (FGM), field goals attempted (FGA) and field goals successful (FG%), offensive rebounds (OR), defensive rebounds (DR), assists (A), turnovers (TO), and steals (S). Variables related to the opponent line-up were also considered, represented with an (o) after the name (e.g., assist by the opponent ‘Ao’). For each five-player composition, functional classes of players on the court were identified and concatenated to generate an LT variable.

A descriptive analysis of the 588 lineups employed during the competition identified seventy distinct LTs. Lineups were classified based on their point differential (outcome) as positive when the total points scored by the lineup exceeded the points conceded, and as negative when the points scored were equal to or fewer than those conceded. Matches were subsequently classified as balanced or unbalanced according to a 12-point score differential threshold, consistent with previous research (Gómez et al., 2015), corresponding to the median points differential observed across the sample.

### Measures

The GRS were normalised, and key performance factors of basketball were calculated (Kubatko et al., 2007; Oliver, 2004), where variables were divided by playing time and multiplied by forty minutes (match time). Subsequently, to ensure data validity, lineups with less than one minute of on-court deployment (n = 61) were removed from the statistical analysis, as they presented substantial missing data that complicated normalisation and comparison (Becerra-Muñoz et al., 2023). To avoid multicollinearity among variables in the discriminant analysis, a collinearity assessment was conducted through the calculation of the variance inflation factor (VIF). This analysis revealed that all variables exhibited VIF values within acceptable ranges (VIF < 2), except for ST (2.51) and TOo (2.94), which showed slightly higher VIF values (George and Mallery, 2024; Ziv et al., 2010). However, these values remained below the commonly accepted threshold (VIF < 5). Given the conceptual relevance of both variables, as not all turnovers result from steals and both capture distinct aspects of game performance, both were retained in the analysis.

A k-means cluster analysis was performed to group the seventy LTs according to the FC of players, aiming to identify similar characteristics between LTs that shared a similar structure in players’ FC composition (George and Mallery, 2024; O'Donoghue and Holmes, 2014).

### Statistical Analysis

For the first objective, a discriminant analysis was performed to identify the variables that best classified the lineups with a final positive/negative outcome in balanced and unbalanced matches. Structural coefficients (SCs) greater than │0.30│ allowed us to identify the variables that best contributed to differentiating the lineups with a positive outcome from those with a negative outcome. The discriminant models were validated by employing an exclusion classification. In addition, cross-validation of the discriminant models was performed using the “leave-one-out” classification method (George and Mallery, 2024).

The normality assumption was assessed using the Kolmogorov-Smirnov test, which indicated significant deviations from normality (*p* < 0.001) across all variables. Consequently, for the second objective, a Kruskal-Wallis test was performed to identify the differences among the game variables by cluster analysis and time window as 1-to-5 min (window 1), 5.01-to-10 min (window 2), and exceeding 10.01 min (window 3) (Alonso et al., 2020; Richardson and Machan, 2021). A pairwise post hoc comparison was then conducted using the Dwass-Steel-Critchlow-Fligner (DSCF) method. For the third objective, descriptive statistics, mean (M) and standard deviation (SD), were calculated for the GRS within specified time windows. The analysis focused on the GRS associated with the most frequently used LT among national teams during the competition. Statistical analysis was carried out using Excel 2019 (Microsoft. Redmond, WA, United States, 2019), IBM SPSS Statistics version 29 (IBM. Armonk, NY, United States, 2022) and Jamovi 2.6.22.0 (JASP Project, Sydney, Australia, 2024). The significance level was set at *p* < 0.05.

## Results

### Discriminant Analysis of Lineups by the Outcome

The discriminant analysis differentiated between lineups with positive and negative outcomes in balanced and unbalanced matches (Table 1). Significant differences were observed in the initial univariate comparisons within the discriminant analysis for balanced matches, particularly in %FG, DR (for and against), AS (for and against), and TO (for and against) (*p* < 0.05) in favour of positive outcome lineups. In unbalanced matches, all GRS showed significant differences (*p* < 0.001) in favour of positive outcome lineups, except for ORo and OR. The most decisive variables for discriminating lineups with positive and negative outcomes in balanced matches (λ = 0.66; CC = 0.59; *p* < 0.001) were %FG (SC = 0.74), DRo (SC = −0.51), AS (SC = 0.65), and ASo (SC = −0.47). In unbalanced matches (λ = 0.47; CC = 0.72; *p* < 0.001), %FG (SC = 0.62), DRo (SC = −0.35), AS (SC = 0.57), and ASo (SC = −0.50) were also the variables that best discriminated between positive and negative outcomes (Table 1). The cross-validation of the discriminant model reported a correct reclassification percentage of 80.1% for balanced matches and 86.6% for unbalanced matches.

**Table 1:** Means (M), standard deviations (SD) and structural coefficients (SC) of lineup game-related statistics with positive and negative results in balanced and unbalanced games at the male WB competition of the Tokyo 2020 Paralympic Games.

Game Statistics	Balanced games					Unbalanced games				
	Positive Result		Negative Result		SC	Positive Result		Negative Result		SC
	M	SD	M	SD		M	SD	M	SD	
% FG^a,c^	52	14.9	30.4	18.2	0.74*	53.7	18.4	29.0	18.0	0.62*
OR	7.9	8	8.7	11.3	−0.001	12.5	13.4	8.0	9.9	0.07
DR^b,c^	31.9	8.6	28.7	13.2	0.21	33.6	12.5	25.9	13.4	0.26
ORo	9.1	9.6	7.9	10.8	0.11	8.8	12.9	11.2	12.6	−0.09
DRo^a,c^	24.4	9	35.3	15.7	−0.51*	24.9	11.1	34.9	13.7	−0.35*
AS^a,c^	26	9.9	15.8	10.9	0.65*	28.2	11.5	14.5	10.0	0.57*
TO^b,c^	8.9	7.7	13.5	14.3	−0.24	8.4	8.5	19.7	27.4	−0.25
ST^c^	5.8	6.6	4.5	7.2	0.13	8.9	10.2	3.6	6.2	0.21
ASo^a,c^	16.5	8.8	24.8	14.2	−0.47*	14.0	10.4	28.3	13.3	−0.50*
TOo^b,c^	12	9.4	9	10.1	0.18	18.1	14.3	9.7	10.4	0.29
STo^c^	4.8	5.9	5.9	10	−0.08	3.5	5.7	8.3	10.3	−0.28

* Values of the discriminant coefficients ≥│0.30│; ^a^ Significant differences in balanced games (p < 0.001); ^b^ (p < 0.05); ^c^ Significant differences in unbalanced games (p < 0.001)

### Analysis of Lineups by the Cluster and the Time Window

**Figure 1:**
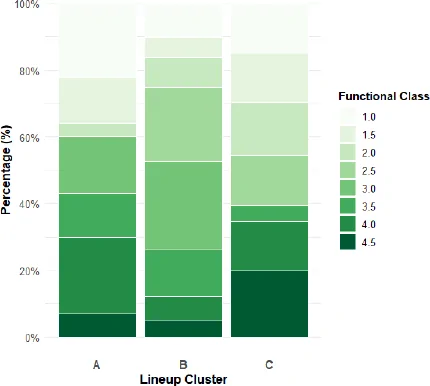
Lineup composition percentage by functional classification across clusters at the male WB competition of the Tokyo 2020 Paralympic Games.

Three LT clusters were identified from the classification performed. Cluster A (n = 20 LTs) primarily comprised LTs mainly composed by 1.0- and 4.0-point players, with few 2.0- and 4.5-point players, and no 2.5-point players; LTs in cluster B (n = 35 LTs) were characterised by a greater presence of 2.5-, 3.0-, and 3.5-point players, and few 4.5-point players, and LTs in cluster C (n = 15 LTs) were mainly composed by 4.5-point players, with few 3.5-point players and no 3.0-point players (Figure 1).

Significant differences were found in LT cluster comparison for variables FGM, ST, and ASo (*p* < 0.05) and significant differences for the time window factor in the variables FGM, FG%, OR, AS, ST (*p* < 0.001), FGAo, ORo, STo, and OR% (*p* < 0.05). Table 2 shows the comparison of means among the three types of clusters for each time window where significant differences were found for variables FGM, ORo, AS, %DR, and %RB in window 1 (*p* < 0.05), only for FGA variable in window 2, and for variables plus/minus, FGM, FGA, AS, TO, ST, ASo, and TOo in window 3 (*p* < 0.05).

**Table 2:** Mean (M) and standard deviations (SD) of performance variables by lineup clusters and time intervals at the male WB competition of the Tokyo 2020 Paralympic Games.

TW	C	N		+/-	FGM	FGA	FG%	OR	DR	ORo	DRo	OR%	DR%	RB%	AS	TO	ST	ASo	TOo	STo
W1	A	96	M	−1.5	19.4	61	33.1	10.3	26.9	6.9	32.3	19.8	82.6	52.1	17.2	15.3	6.3	27.1	12.3	6.4
			SD	3.9	15.9	28	25.8	21.4	17.8	12.6	19.3	27.7	28	19.9	15.2	17.7	11.1	18.2	15.3	10.1
	B	135	M	−0.3	25.6*	63.5	40.5	9.4	29.2	10.6*	30.2	22.7	77.3	50	20.3*	14.5	6.5	22.6	13.1	6.3
			SD	4	19	18.9	28.1	12.7	19.2	14.5	18.5	27.6	26	18.7	16.7	14.6	10.5	15.6	15.3	10.5
	C	66	M	−1.1	20.9	57.9	36.6	6.9	28.8	13.4*	31.5	15.1	70.8	43.1	17	17.9	4.3	21.1	11.7	9.1
			SD	3.5	17.5	22.2	31.4	10	17.7	17.7	19.9	21.3	32	17.4	14.4	19.8	8.8	17.7	13.8	14.9
W2	A	50	M	0.4	26.2	59.8	44.3	7.1	29.6	7.9	27.7	20	80.3	50.1	22	12.8	5	21.4	11.8	5.5
			SD	6.1	10.7	9.1	17.7	6.4	8.9	7.3	11	17.2	17.3	11.7	9.7	9.6	6.6	9.3	9.7	5.9
	B	67	M	1.3	27.9	65*	44.1	9.6	29.3	7.5	29.5	21.5	81.3	51.4	22.1	14.5	6.3	19.3	13.8	4.8
			SD	5.6	9.3	12.6	16	9.3	7.6	6.4	10.5	17.2	14.2	10.6	9.1	33.1	5.4	10.3	9.1	5.3
	C	29	M	0.6	27.7	66.3*	42.1	11.9	27.8	7.4	29	25.5	80.8	53.2	22	11.4	5.7	20.8	10.6	5.3
			SD	7.5	10.5	12.9	14.1	11.7	9.5	8.1	9.3	21.8	21.1	13.2	9.6	8	6.5	10.7	9.6	5.9
W3	A	23	M	−3.1	24.3	59.1	41.1	7.4	26.9	9.2	29	20.1	75.6	47.8	20.6	13.1*	5*	22.7	10.8*	6.2
			SD	10	7.5	7.4	11.4	4.8	5	5.9	6.8	11.7	14.4	10.8	7.6	5.3	4.4	6.3	5.6	4.4
	B	39	M	5.2*	29.7*	64*	46.4	10.2	28.2	8	27	24.1	78.1	51.1*	24.7*	9.9	6.2*	17.7*	13.2*	4.5*
			SD	10.2	6.8	7.9	9.1	14.9	9.6	6.9	7.3	16.1	16.8	9.3	8.5	5	4.2	7.4	6.1	3.5
	C	22	M	0.5	28,3	63,6*	44.4	7.8	29.2	7	28.5	21	80.8*	50.9*	21.1	7.8	3.1	23.3	7.7	3.9*
			SD	9.7	6,9	7,1	9.5	4.6	5.7	4.3	5.3	10.5	10.6	7.6	5.3	4.6	3.1	6.9	5.7	2.9

TW: Time windows 1–5 min (W1), 5–10 min (W2) and exceeding 10 min (W3); C: cluster lineup types; ^*^ Significant differences by the interval time in the pairwise post hoc comparison (p < 0.05)

### Descriptive Analysis of Team Performance

The 144 players participating in the WB male competition at the Tokyo 2020 Paralympic Games, classified by the team and FC, are detailed in Table 3. Each national team had 12 players. Japan and Colombia were the only teams that included players from all FC classes in their rosters, while Australia and Germany had players available from four FC classes only. Colombia and Australia were the teams that used the most LTs from cluster A, the United States and Japan (both finalists at this competition) were the teams that used the most LTs from cluster B, and Iran was the team that used the most LTs from cluster C. The FC class that had the most players available during the tournament were 4.0 (25 players), 3.0 (22 players), and 1.0 (22 players). Germany, Canada, and Iran had a greater number of 1.0- to 2.0-point players available on their rosters, while Spain, the United States, Japan, Great Britain, Colombia, and Algeria had a greater number of 2.5 to 3.5-point players available. Finally, Australia, Turkey, and Korea showed the same number of players available for low, intermediate, and high FC.

**Table 3:** Team Player Availability per Functional Class at the male WB competition of the Tokyo 2020 Paralympic Games, with indication of the total and LT type used per team.

Team	Functional Classification								TP	FR	LTs A	LTs B	LTs C	TLTs
	1.0	1.5	2.0	2.5	3.0	3.5	4.0	4.5						
USA	2	0	1	2	1	3	1	2	12	1	2	10	0	12
JPN	1	1	2	2	1	2	2	1	12	2	2	10	3	15
GBR	2	0	2	1	3	1	1	2	12	3	3	6	2	11
ESP	1	2	0	2	4	0	2	1	12	4	3	5	0	8
AUS	2	2	0	0	4	0	4	0	12	5	7	3	0	10
TUR	2	1	1	2	2	0	3	1	12	6	3	6	2	11
GER	3	0	2	0	4	0	0	3	12	7	0	2	1	3
CAN	2	3	1	1	0	2	0	3	12	8	2	0	3	5
IRI	2	1	2	1	0	2	3	1	12	9	4	0	5	9
KOR	2	0	2	2	1	1	4	0	12	10	3	4	3	10
COL	2	1	1	1	1	3	1	2	12	11	7	0	3	10
ALG	1	1	1	2	1	2	4	0	12	12	3	5	1	9

TP: total players; FR: final rank; LTs(x): Number of lineup types used by the team by the LT cluster; TLT: Total number of lineup types used by the team

We can highlight those LTs that performed prominently for a given team/country along the different time windows. Regarding the performance of the most used LTs by the teams along the different time windows, it was observed that the United States with LT 37 (1-2.5-2.5-3.5-4.5) had the best performance for the TOo variable within time window 3, ST within windows 2 and 3, and plus/min and %RB within window 3. Likewise, Germany had the best performance with LT 58 (1-2-3-3-4.5) regarding TO, STo, and %DR within window 2 and also in ASo and %OR within window 3. On the other hand, Japan with LT 12 (1.5-2-2.5-3.5-4.5) had the best performance in the FG% variable within windows 1 and 3 and with LT 65 (2-2.5-2.5-3.5-3.5) in FG% and ASo in window 2. Great Britain with LT 56 (1-2-3-3.5-4.5) stood out in DR% within window 1 and plus/minus, AS, OR%, and %RB within window 2. Also, Australia had the best performance with LT 62 (1-3-3-3-4) in the AS variable within windows 1 and 3 and in plus/minus and ASo within window 1.

Lastly, Spain had the best performance with LT 10 (1.5-2.5-3-3-4) in %RB within window 3; Turkey also stood out with LT 10 in STo within window 1; Canada had the lowest value in STo with LT 19 (1-1.5-2.5-4.5-4.5); Iran with LT 32 (1-1-3.5-4-4.5) had the best performance in %OR within window 1 and with LT 51 (1-2-2.5-4-4.5) in TO within window 3; Algeria with LT 60 (1-3-3.5-3.5-4) had the best performance in TO and ST within window 1; Korea stood out in %RB with LT 59 (1-2-3-4-4) within window 1; and finally, Colombia, mostly used LT 56 (1-2-3-3.5-4.5), but did not have the highest average in any game-related variable.

## Discussion

This study analysed variables influencing success during the WB competition at the 2020 Tokyo Paralympic Games, focusing on lineup composition by FC and time windows. This approach is novel, as research is scarce, particularly regarding lineup and time window analysis in top male WB competition. Consequently, this study will draw upon discussions of individual player performance within the functional classification framework and recent team performance articles, due to the limited existing literature on WB lineup analysis. Results from the first objective suggested that achieving a positive outcome in men's competition requires strong performance in AS and %FG (Francis et al., 2019), coupled with proficiency in DR. However, unlike previous studies that identified ST (Gómez et al., 2014; from an individual GRS analysis) as a key variable, this analysis identified minimising opponent assists (ASo) as a determinant of the point outcome, which puts value on defensive aspects at this level of performance when it comes to causing both the opponent's losses / increasing the number of ST and negatively influencing the offensive development of the opponent team, by reducing the number of AS. Notably, and in contrast to the women's competition (Becerra-Muñoz et al., 2023), no differences were observed in the key variables determining success between balanced and unbalanced matches.

Prior research has indicated that the use of intermediate point players (2.0-, 2.5-, 3.0- and 3.5-point players) is a common trend in actual WB lineup composition due to their physical attributes and GRS performance (Molik et al., 2009; Pérez-Tejero and Pinilla-Arbex, 2015; Vanlandewijck et al., 2004). This observation is supported by the classification clusters identified in this study, where cluster B lineups were characterised by predominance of these intermediate FC class players, demonstrating superior %FG across all three time windows. These lineups also excelled significantly in AS, TO, and ST (both for and against) during window 3 (more than 10 min; Table 2). These findings align with those reported by Francis et al. (2019), which identified lineups composed mainly of intermediate FC players as optimal during competition, exhibiting a superior balance between offensive and defensive efficiency. In this regard, the players’ distribution of LTs within Cluster B is consistent with the findings reported in the same study, where optimal odds ratios for match success were identified for a lineup comprising one 1.0–1.5 point player, one 2.0–2.5 point player, two 3.0–3.5 point players, and one 4.0–4.5 point player. These findings are further supported by the present analysis, in which we observed that teams with higher final ranking utilised intermediate FC lineups (Cluster B) more frequently, providing evidence of the association between this lineup composition and positive match outcomes.

Consistent with previous findings, Yasuda et al. (2024), in a performance analysis also about the Paralympic Games from individual GRS, demonstrated that 4.0- and 4.5-point players continued to excel in scoring 2- and 3-point field goals with high %FG in winning teams. However, it was also further revealed that intermediate point players were instrumental in winning teams, particularly within the 3-point zone, as they executed the highest percentage of offensive screens and provided AS for successful 2- and 3-point field goal attempts. Additionally, in the context of the United States team (Arroyo et al., 2023), these intermediate point players exhibited a high frequency of shooting attempts from various offensive court positions. Although a notable portion of these attempts correlated with reduced shooting efficiency, it is important to note that the United States´ team occasionally attempted a greater volume of field goals than their opponents did, even with lower efficiency, which was evidenced by their intermediate point players, a trend similarly observed in the results from the Japanese team in this study. Discussing about the different teams´ results, analysis of frequently used lineups across time windows indicates that top- ranked teams consistently achieved superior GRS performance. Notably, the United States and Japanese teams demonstrated the highest average GRS values across multiple time windows, supporting the link among the final ranking position (Gómez et al., 2015; Molik et al., 2009), tactical performance (Francis et al., 2019), and players’ roles (Vanlandewijck et al., 2004). Japan excelled in %FG across all time windows, while the United States outperformed in GRS such as AS, TO, and ST (for and against). These key GRS, where top-ranked teams excelled, align with those discriminating between winning and losing lineups (Becerra-Muñoz et al., 2023) and those differentiating among performances in Cluster B lineups. Conversely, it is notable that Spain and GBR, despite competing for the bronze medal, did not exhibit superior performance in the variables associated with lineup success, instead excelling primarily in OR, DR, and RB%.

Furthermore, in our study, it was observed that both roster depth and the use of a greater variety of LTs were characteristics of the gold medal-winning team and other top-ranked teams in the men's competition, similarly to the women's competition in this sport event (Becerra-Muñoz et al., 2023). Although, in that study, it was interesting to note that the trend showed a greater use of low (1.0 and 1.5) and high (4.0 and 4.5) FC points players in the composition of LTs for the women’s competition, indicating a marked difference from the findings of the present study, where intermediate point players were way more present in the different LTs used. Conversely, teams that infrequently or did not use Cluster B lineups tended to have lower final rankings, often coupled with limited player availability within certain FCs and a smaller repertoire of LTs (Table 3).

While some GRS differences favouring Cluster B were observed along time windows 1 and 2, the most pronounced differences emerged along window 3 (over 10 min of play), where Cluster B lineups outperformed Clusters A and C across most of the variables. This suggests that, although considerable substitutions are evident along the WB match, game situations usually last around 30 s (Pérez-Tejero et al., 2020) and periods of maximum demand occur at specific times (Fox et al., 2021; Pérez-Chao et al., 2023). Therefore, identifying effective lineups capable of delivering high performance, for a quarter or more, is crucial to team success, especially when combined with strategically varied lineup rotations. In this context, Clay and Clay (2014) highlighted the impact of rotations on team performance in running basketball, finding that, for offensive tactics, maintaining specific lineups and a lower number of substitutions seemed to yield better results in shooting efficiency, ball control, and offensive performance. Conversely, for defensive tactics, being able to substitute players and having “fresh players” to win defensive rebounds, make steals, and perform efficiently in defence is advantageous. In this regard, future research should consider not only differences related to functional classification and players’ positions when analysing lineups and rotation strategies, but also the on-court performance between starters and bench players (Gonçalves et al., 2025; Martinho et al., 2025). As Sun et al. (2023) pointed out, a characteristic of top-performing teams largely depends on minimal differences in on-court performance between starting players and those on the bench. These observations might explain why lineups have been used for extended times combined with multiple variations of LTs; however, it is necessary to delve deeper into the study of moving averages, most demanding scenarios, and play-by-play analysis to determine how substitutions might impact performance of a given LT.

The methodological constraints of this study limited the ability to accurately identify the specific moments of lineup deployment during matches, thereby hindering a comprehensive assessment of their influence on match outcomes. Additionally, the decision to exclude lineups with less than one minute of playing time potentially overlooked crucial high-demand scenarios. Another limitation is the absence of positional data for players within lineups. The analysis did not account for potential variations in player positions within the same lineup, which could significantly impact performance dynamics. Although this GRS analysis offers significant insights into lineup performance, future research should consider incorporating substitution patterns, contextual game variables, players’ roles within specific lineups, play-by-play data, and team tactical strategies to enhance our understanding of team performance and lineup composition strategies.

## Conclusions

Key determinants of success in men's Paralympic WB at the 2020 Tokyo Games from lineup analysis included high field goal efficiency, maximising assists, and minimising opponent assists (effective defence) and opponent offensive rebounds, regardless of game balance. Furthermore, lineup composition strategies that incorporate players from intermediate point players (2.0–3.5) and utilise a diverse range of lineup types may enhance team performance. Specifically, extended playing time (over 10 min) with lineups predominantly composed of these intermediate point players appears to be related to improved GRS compared to shorter duration or alternative lineup configurations. These findings offer valuable insights for coaches and staff, enabling data-driven decisions regarding lineup selection and strategic planning to optimise both pre-competition preparation and in-game adjustments.
